# Characterization of the biofilm phenotype of a *Listeria monocytogenes* mutant deficient in *agr* peptide sensing

**DOI:** 10.1002/mbo3.826

**Published:** 2019-03-06

**Authors:** Marion Zetzmann, Florentina Ionela Bucur, Peter Crauwels, Daniela Borda, Anca Ioana Nicolau, Leontina Grigore‐Gurgu, Gerd M. Seibold, Christian U. Riedel

**Affiliations:** ^1^ Institute of Microbiology and Biotechnology University of Ulm Ulm Germany; ^2^ Faculty of Food Science and Engineering Dunarea de Jos University of Galati Galati Romania

**Keywords:** biofilm, cell envelope, *Listeria monocytogenes*, peptide sensing, stress

## Abstract

*Listeria monocytogenes* is a food‐borne human pathogen and a serious concern in food production and preservation. Previous studies have shown that biofilm formation of *L. monocytogenes* and presence of extracellular DNA (eDNA) in the biofilm matrix varies with environmental conditions and may involve *agr* peptide sensing. Experiments in normal and diluted (hypoosmotic) complex media at different temperatures revealed reduced biofilm formation of *L. monocytogenes* EGD‐e Δ*agrD*, a mutant deficient in *agr* peptide sensing, specifically in diluted Brain Heart Infusion at 25°C. This defect was not related to reduced sensitivity to DNase treatment suggesting sufficient levels of eDNA. Re‐analysis of a previously published transcriptional profiling indicated that a total of 132 stress‐related genes, that is 78.6% of the SigB‐dependent stress regulon, are differentially expressed in the Δ*agrD* mutant. Additionally, a number of genes involved in flagellar motility and a large number of other surface proteins including internalins, peptidoglycan binding and cell wall modifying proteins showed *agr*‐dependent gene expression. However, survival of the Δ*agrD* mutant in hypoosmotic conditions or following exposure to high hydrostatic pressure was comparable to the wild type. Also, flagellar motility and surface hydrophobicity were not affected. However, the Δ*agrD* mutant displayed a significantly reduced viability upon challenge with lysozyme. These results suggest that the biofilm phenotype of the Δ*agrD* mutant is not a consequence of reduced resistance to hypoosmotic or high pressure stress, motility or surface hydrophobicity. Instead, *agr* peptide sensing seems to be required for proper regulation of biosynthesis, structure and function of the cell envelope, adhesion to the substratum, and/or interaction of bacteria within a biofilm.

## 1 INTRODUCTION

*Listeria monocytogenes* is a saprophytic soil organism that is widespread in nature (Vivant, Garmyn, & Piveteau, 2013) and frequently found in food processing environments posing a threat to the food chain (Bolocan et al., ; Muhterem‐Uyar et al., 2015; NicAogáin & O'Byrne, 2016). In healthy individuals, food‐borne infections with *L. monocytogenes* result in mild gastroenteritis or remain completely asymptomatic. However, in at‐risk groups such as immunocompromised persons, elderly people and pregnant women, *L. monocytogenes* may cause life‐threatening disease (Allerberger & Wagner, 2010; Vázquez‐Boland et al., 2001).

Two characteristics that make *L. monocytogenes* a major concern in food processing and sanitation of the respective production lines are the ability to form surface‐attached communities (also referred to as biofilm formation) and an extremely high tolerance to a wide range of environmental conditions and stresses (Ferreira, Wiedmann, Teixeira, & Stasiewicz, 2014; NicAogáin & O'Byrne, 2016).

Following initial adhesion, *L. monocytogenes* is able to form surface‐attached communities (Carpentier & Cerf, 2011; Renier, Hébraud, & Desvaux, 2011; da Silva & De Martinis, 2013). The population density in these communities is 1–2 orders of magnitude lower than that observed for surface‐attached communities of other bacteria (da Silva & De Martinis, 2013). Compared to other bacteria, biofilm formation of *L. monocytogenes* is not as pronounced, but may be enhanced by precolonization of surfaces by other bacteria such as *Pseudomonas putida* and *Flavobacterium sp*., probably involving the extracellular polymeric substances (EPS) produced by these bacteria (Giaouris et al., 2015). By contrast, precolonization of surfaces with, for example, *Pseudomonas fragi* and *Serratia ssp*. reduced biofilm formation of *L. monocytogenes*. There are conflicting results regarding the production of EPS by *L. monocytogenes*. Some studies conclude that *L. monocytogenes* biofilms generally lack EPS (Renier et al., 2011). By contrast, a recent study could show that EPS production by *L. monocytogenes* can be induced by elevated levels of the second messenger cyclic di‐GMP and the genetic locus for EPS production was identified (Chen et al., 2014). This leaves room for interpretation as to whether or not these communities are biofilms according to the strict definition, which requires the communities to be embedded into a self‐produced matrix of extracellular polymeric substances (Flemming & Wingender, 2010). Nevertheless, several studies have provided evidence for three‐dimensional structures described as honey‐comb or knitted chains and the presence of extracellular DNA (eDNA) and exopolysaccharides (Borucki, Peppin, White, Loge, & Call, 2003; Guilbaud, Piveteau, Desvaux, Brisse, & Briandet, 2015; Harmsen, Lappann, Knøchel, & Molin, 2010; Rieu et al., 2008; Zetzmann et al., 2015). Thus, it seems reasonable to consider surface attached communities of *L. monocytogenes* as biofilms.

The accessory gene regulator (*agr*) locus encodes for a peptide sensing system that is found in many Gram‐positive organisms and has pleiotropic effects (Wuster & Babu, 2008). For *L. monocytogenes*, it has been shown that the *agr* system is involved in the regulation of virulence and biofilm formation (Autret, Raynaud, Dubail, Berche, & Charbit, 2003; Riedel et al., 2009; Rieu, Weidmann, Garmyn, Piveteau, & Guzzo, 2007) and is required for survival in soil (Vivant, Garmyn, Gal, Hartmann, & Piveteau, 2015). Recently, the native autoinducing peptide of the *L. monocytogenes agr* system has been identified (Zetzmann, Sánchez‐Kopper, Waidmann, Blombach, & Riedel, 2016).

The aim of this study was to investigate the biofilm phenotype of a *L. monocytogenes* mutant deficient in *agr* peptide sensing.

## 2 MATERIALS AND METHODS

### 2.1 Bacterial strains and growth conditions

In this study, *L. monocytogenes* strains EGD‐e, its isogenic mutant EGD‐e Δ*agrD*, and the genetically complemented strain EGD‐e Δ*agrD*::pIMK2*agrD* were used. All strains have been described previously (Riedel et al., 2009). Bacteria were cultivated routinely in brain heart infusion broth (BHI, Oxoid, Altrincham, Cheshire, England) or 10‐fold diluted BHI (0.1BHI) at 25 or 37°C. Precultures for functional assays were prepared by inoculation of a single colony from a fresh agar plate into 10 ml BHI and incubated aerobically on a rotary shaker (200 rpm) at 25°C overnight (o/N, i.e., approx. 16 hr).

### 2.2 Quantification of surface‐attached biomass

To quantify surface‐attached biomass, classical crystal violet assays were performed in 96‐well microtiter plates as described previously (Zetzmann et al., 2015). Where indicated, 1 unit (U) of DNase I (Thermo Scientific, Waltham, MA) or 1 mg/ml pronase (Sigma‐Aldrich, Darmstadt, Germany) was added to the wells directly after inoculation. Plates were incubated at 25°C or 37°C for 24 hr. For analysis, biofilms were washed gently twice with phosphate‐buffered saline (PBS) followed by staining with 0.1% (v/v) crystal violet solution (Merck, Darmstadt, Germany) for 30 min. After three further washings with PBS, crystal violet was released from biofilms by addition of 100 µl 96% (v/v) ethanol and incubated for 10 min. Biofilm biomass was quantified by measuring absorbance at 562 nm (Abs_562 nm_) with background correction, that is, crystal violet staining in wells incubated with sterile media under the same conditions.

### 2.3 Membrane and cell wall stress assays

To assess the effects of reduced osmolarity in 0.1BHI on viability of bacteria, aliquots of the preculture used for biofilm assays were diluted 1:100 in either 0.1BHI or demineralized H_2_O (dH_2_O) and viable cell counts were determined as colony‐forming units per ml (CFU/ml) by spot‐plating. For this purpose, 10 µl aliquots of 10‐fold serial dilutions were plated in triplicate onto BHI agar and the colonies of an appropriate dilution were counted to calculate CFU/ml. The effect of lysozyme treatment was analyzed in a similar assay except that bacteria were inoculated from a preculture into 0.1BHI, grown at 25°C to exponential growth phase (OD_600nm_ = 0.15–0.2), harvested by centrifugation and resuspended in 0.1BHI containing 5 µg/ml lysozyme (40,000 Units/mg, Sigma‐Aldrich). Bacteria were incubated in the presence of lysozyme at 25°C for the indicated time and log‐reduction was calculated relative to CFU/ml at *t* = 0 min of an untreated control, that is, an aliquot resuspended in 0.1BHI without lysozyme.

### 2.4 High hydrostatic pressure treatments

For high hydrostatic pressure (HPP) experiments, a single colony from a fresh BHI agar plate was inoculated into BHI broth and grown for 12 hr at 37°C. This preculture was diluted to an OD_600nm_ of 0.05 in 0.1BHI and grown for 1.5–2 hr to exponential growth phase (i.e., OD_600nm_ of 0.15 ± 0.02). At this stage, samples of 2 ml were loaded in Eppendorf tubes and sealed by carefully avoiding any air bubbles inside. Pressure treatments were conducted in a multivessel (four vessels of 100 ml) high‐pressure equipment (Resato, Roden, the Netherlands) at 20 ± 0.5°C. As a pressure transmitting fluid a mixture of water and propylene glycol fluid (TR15, Resato) was used. Pressure treatments were performed at 200, 300, and 400 MPa with a compression rate of 250 MPa/min and 60 s after the come‐up time were considered the equilibration time necessary for each treatment. Samples were maintained for an additional 60 s at the established pressure followed by decompression of the vessels in less than 5 s. Treated samples were removed from the high‐pressure vessels, and immediately afterwards, viable cell counts (CFU/ml) were quantified by spot‐plating as described above.

### 2.5 Motility assays

To assess motility of bacteria, precultures were prepared as described above in 0.1BHI at 25°C o/N. Of these precultures, soft agar of the same medium (0.1BHI, 0.2% agar) were inoculated by dipping an inoculation needle in the preculture and briefly stabbing onto the surface of the soft agar plate. After incubation for 24 h at 25°C, plates were imaged using a standard digital camera and the size of the zone of growth around the spot of inoculation was measured.

### 2.6 Microbial adhesion to hydrocarbons

Surface hydrophobicity of all strains was evaluated using a standard assay to quantify microbial adhesion to hydrocarbons (MATH assay) (Rosenberg, 2006). Briefly, bacteria were grown in 0.1BHI at 25°C o/N, washed once in PBS and adjusted to an OD_600nm_ of 0.1 in PBS (OD1). Two milliliters of this suspension were mixed with 0.4 ml xylene and vortexed for 2 min. After separation of the phases, OD_600nm_ was again measured in the aqueous phase (OD2). Hydrophobicity (*H*) was then calculated as $\left[\%\right]=\frac{\left(OD1-OD2\right)}{OD1}\times 100$ .

### 2.7 Statistical analysis

Statistical analysis was performed by Student's *t* test or analysis of variance (ANOVA) with Dunnett's posttest to adjust *P*‐values for multiple comparisons using GraphPad Prism (version 6). Differences were considered significant at *p* < 0.05.

## 3 RESULTS AND DISCUSSION

**Figure 1:**
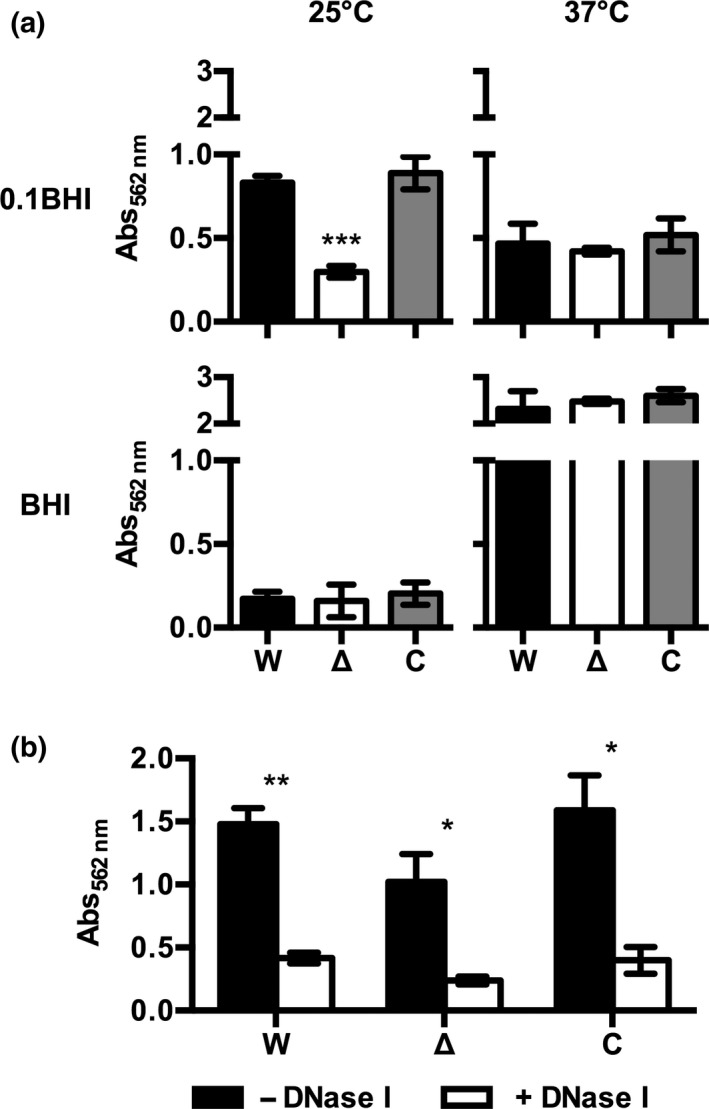
Biofilm formation (a) and DNAseI sensitivity of biofilms (b) of *L. monocytogenes* EGD‐e WT (W), EGD‐e Δ*agrD* (Δ), and EGD‐e Δ*agr*D::pIMK2*agrD* (C). Biofilms were grown in BHI or 0.1BHI at 25 or 37°C in the absence (a) or presence of DNaseI (b; 0.1BHI at 25°C only). Biofilm biomass was quantified by crystal violet staining and measuring absorbance at 562 nm (Abs_562nm_) after 24 hr of growth in polystyrene microtiter plates. All values are mean ± standard deviation of three independent experiments. Statistical analysis was performed by ANOVA with Dunnett's multiple comparisons test with *L. monocytogenes* EGD‐e WT set as control condition (a) or Student's *t* test comparing biofilm of each strain in the presence and absence of DNase I (b; *p < 0.05; **p < 0.01; ***p < 0.001)

Recently, we were able to show that biomass and presence of eDNA in biofilms of *L. monocytogenes* EGD‐e vary with growth conditions (Zetzmann et al., 2015). Additionally, a *L. monocytogenes* EGD‐e Δ*agrD* deletion mutant showed reduced levels of surface‐attached biomass in 0.1BHI at room temperature (Riedel et al., 2009). In order to investigate if *agr* peptide sensing is a general regulatory mechanism in formation of surface‐attached bacterial communities by *L. monocytogenes* or only relevant under specific conditions, biofilm formation of *L. monocytogenes* EGD‐e wildtype (WT), *L. monocytogenes* EGD‐e Δ*agrD*, and the genetically complemented strain *L. monocytogenes* EGD‐e Δ*agrD*::pIMK2*agrD* were assessed following growth in static culture in microtiter plates in full strength and 0.1BHI at 25 and 37°C (Figure 1a). In line with our previous study, highest biofilm levels of all strains were observed in BHI at 37°C followed by 0.1BHI at 25°C and lowest biofilm biomass was formed in 0.1BHI at 37°C. Interestingly, the Δ*agrD* mutant showed reduced biofilm formation only in 0.1BHI at 25°C. For all other conditions, no difference was observed between the three strains. Thus, *agr* peptide sensing is required for proper regulation of biofilm formation under specific conditions, that is, in 0.1BHI at 25°C.

Interestingly, these are the conditions under which biofilms of the WT strain showed increased abundance of eDNA and DNase I sensitivity (Zetzmann et al., 2015). This prompted us to test whether loss of *agr* peptide signaling is associated with altered sensitivity toward DNase I treatment (Figure 1b). However, biofilm formation of the Δ*agrD* mutant was reduced by DNase I to a similar extent as observed for the WT (and complemented strain) at 25°C in 0.1BHI indicating that eDNA is present in these communities and lack of eDNA is not responsible for the observed phenotype of *L. monocytogenes* EGD‐e Δ*agrD*.

Since the conditions that produce the phenotype of Δ*agrD* mutant may cause osmotic stress due to the low nutrient and ion concentration in dH_2_O‐diluted BHI (0.1BHI). In a previous study, a deletion mutant in the AgrC sensor histidine kinase of the *agr* system displayed increased sensitivity to high concentrations of salt (Pöntinen, Lindström, Skurnik, & Korkeala, 2017). Thus, we hypothesized that a reduced resistance to osmotic stress may lead to increased lysis of bacteria and, consequently, reduced surface‐attached biomass.

In order to get a first indication whether deletion of *agrD* results in reduced stress resistance, we re‐analyzed a previously published transcriptomic data set comparing *L. monocytogenes* EGD‐e Δ*agrD* with its parental WT strain (Riedel et al., 2009). The conditions of biofilm formation (0.1BHI, 25°C) and the transcriptomic analysis (BHI, 37°C) are different. Nevertheless, we reasoned that the transcriptional data would provide first indications as to whether or not stress related genes are affected by the lack in *agr* peptide signaling and any stress‐related phenotype would be even more evident under for example, hypoosmotic stress (i.e., 0.1BHI). We therefore compared the differentially expressed genes to the regulon of the alternative sigma factor σ^B^, that is, the major regulator of the general stress response in many gram‐positive bacteria including *L. monocytogenes* (Chaturongakul, Raengpradub, Wiedmann, & Boor, 2008; Kazmierczak, Mithoe, Boor, & Wiedmann, 2003; Raengpradub, Wiedmann, & Boor, 2008; van Schaik & Abee, 2005). In *L. monocytogenes*, the σ^B^ regulon comprises 168 genes that are positively regulated by σ^B^ (Raengpradub et al., 2008). Comparison with the 715 genes differentially expressed in *L. monocytogenes* EGD‐e Δ*agrD* revealed an overlap of 132 genes, which is 78.6% of the σ^B^ regulon and 18.5% of the *agr* regulated genes (Table 1 and Supplementary File S1). Of note, only 15 of 132 genes of the overlap show divergent regulation (Supplementary File S1). In other words, 117 genes or 69.6% of the σ^B^ regulon are regulated in the same direction in Δ*sigB* and Δ*agrD* mutants. Thus, deficiency in *agr* peptide sensing results in differential expression of a remarkably large number of genes connected to response and resistance to stress. Similar observations were made previously with a Δ*agrA* mutant that showed reduced expression of several genes under direct positive control of σ^B^ (Garmyn, Augagneur, Gal, Vivant, & Piveteau, 2012).

**Table 1:** Overlap between *agr*‐regulated genes with the SigB stress regulon of *L. monocytogenes*

Group of genes	No. (%) of genes in genome	% of SigB regulon	% of *agr*‐regulated
All in genome^1^	2,587		
*agr*‐regulated^2^	715 (25.0)		
SigB regulon^3^	168 (5.9)		
*agr*‐regulated of sigB regulon	132 (4.6)	78.6	18.5

Notes

Information taken from: ^1^ *L. monocytogenes* EGD‐e genome on Listeriomics database (https://listeriomics.pasteur.fr/Listeriomics/#bacnet.Listeria); ^2^Riedel et al. (2009); ^3^Raengpradub et al. (2008).

Amongst the stress‐related genes differentially expressed in *L. monocytogenes* EGD‐e Δ*agrD* are a number of genes that encode for proteins previously shown to be involved in resistance to cell wall and osmotic stress (Supplementary File S1). For example, *lmo1421*, *lmo1425*, *lmo1426*, and *lmo1427* encode for components of the ABC transporters OpuA and OpuC, which are required for transport of the compatible solutes glycine/betaine, carnitine, and ornithine and were shown to be important for resistance to high salt concentrations (Wemekamp‐Kamphuis et al., 2002). These genes are down‐regulated in both the Δ*agrD* and the Δ*sigB* mutant (Supplementary File S1). In *Escherichia coli*, the glycine/betaine ABC transporter OpuC is also implicated in resistance to HPP used for end‐product decontamination of certain processed foods (Gänzle & Liu, 2015). Other *sigB*‐dependent genes down‐regulated in the Δ*agrD* mutant implicated in the response to osmotic stress are *lmo0555* (encoding a di‐ and tripeptide permease), *lmo2085* (encoding a peptidoglycan‐binding protein), *lmo0232* (encoding the general stress response protein ATPase ClpC), *lmo1879* (encoding a cold‐shock protein) (Burgess et al., 2016), and *lmo2064* (encoding a large conductance mechanosensitive channel protein) whose homologues in various bacteria act as emergency valves that regulate turgor pressure by assisting efflux of osmolytes in response to osmotic down‐shock (Booth, 2014; Booth, Blount, Bootha, & Blount, 2012; Cox, Bavi, & Martinac, 2018). Collectively, this indicates that deletion of *agrD* may impair stress resistance of *L. monocytogenes*.

**Figure 2:**
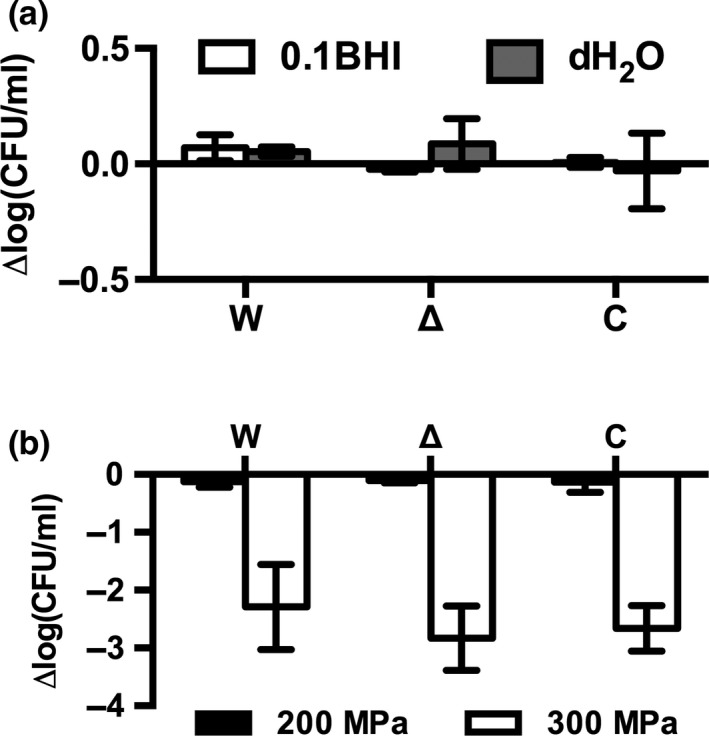
Resistance of *L. monocytogenes* EGD‐e WT (W), EGD‐e Δ*agrD* (Δ), and EGD‐e Δ*agr*D::pIMK2*agrD* (C) exposed to hypoosmotic conditions (a) or high hydrostatic pressure (b). (a) Bacteria were transferred to 0.1BHI or demineralized H_2_O (dH_2_O) and viability was assessed after 60 min by determining CFU/ml. (b) Bacteria from exponential growth phase were resuspended in 0.1BHI and subjected to HPP at the indicated pressure. Changes in viability are reported as Δlog_10_(CFU/ml) compared to bacterial counts before treatment. Values are mean ± standard deviation of three independent experiments. Statistical analysis was performed by ANOVA with Dunnett's multiple comparisons test with *L. monocytogenes* EGD‐e WT set as control condition

In order to test if the changes in expression of stress‐related genes translate to an increased sensitivity of the Δ*agrD* mutant to osmotic stress, survival experiments were performed in 0.1BHI and dH_2_O. This revealed that, after transfer to 0.1BHI or dH_2_O, viable counts (colony forming units) of the mutant, *L. monocytogenes* EGD‐e and EGD‐e Δ*agrD*::pIMK2*agrD* remained constant for at least 1 hr (Figure 2a) suggesting that the phenotype of the Δ*agrD* mutant is not associated with increased lysis in hypoosmotic conditions. In order to corroborate these findings we subjected bacteria to high pressure processing following transfer to 0.1BHI. However, treatment at 200 and 300 MPa was tolerated to a similar extent by the WT, mutant, and complemented strain (Figure 2b) and higher pressure (400 MPa) resulted in complete loss of viability of all three strains (data not shown). This indicates that impaired resistance to osmotic or cell wall stress are not responsible for the reduced biofilm formation of *L. monocytogenes* EGD‐e Δ*agrD*. However, resistance to other stresses (heat, pH, hyperosmotic conditions, etc.) that are not relevant for biofilm formation under the tested conditions may still be affected.

Recently, a Himar transposon mutant library screen identified several genes that are involved in biofilm formation of *L. monocytogenes* 10403S (Alonso, Perry, Regeimbal, Regan, & Higgins, 2014). All of the 38 genes identified in the transposon mutant screen had homologues in *L. monocytogenes* EGD‐e. However, only 6 genes identified in the transposon mutant screen were amongst the genes differentially regulated in *L. monocytogenes* EGD‐e Δ*agrD*. These genes were: *lmo0677* (*fliQ*; homologue of *lmrg_00365*), *lmo0685* (*motA*; *lmrg_00373*), *lmo0973* (*dltB*; *lmrg_02072*), *lmo0974* (*dltA*; *lmrg_02073*), *lmo1768* (*purF*; *lmrg_02503*), and *lmo2785* (*kat*; *lmrg_01912*). With respect to the biofilm phenotype of the Δ*agrD* mutant, especially the identification of genes of the *dlt* operon and the flagellar apparatus are intriguing.

The four genes of the *dltABCD* operon are required for D‐alanine esterification of teichoic acids in the cell wall of *L. monocytogenes*, which is involved in adhesion and virulence (Abachin et al., 2002), and a Δ*dltABCD* mutant showed impaired biofilm formation (Alonso et al., 2014). The entire *dlt* operon was differentially expressed in *L. monocytogenes* EGD‐e Δ*agrD*. However, since its expression was increased in the Δ*agrD* mutant compared to the WT and it was thus ruled out as being responsible for the biofilm phenotype of the mutant.

**Figure 3:**
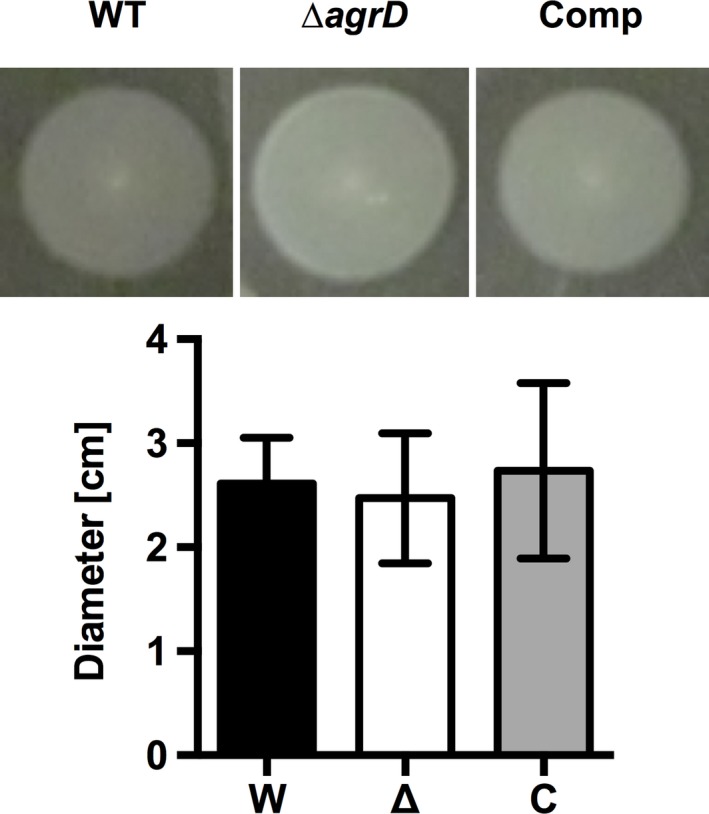
Motility of *L. monocytogenes* EGD‐e WT (W), EGD‐e Δ*agrD* (Δ), and EGD‐e Δ*agr*D::pIMK2*agrD* (C). Representative images and quantification of the diameter of the zone of growth around the inoculation spot of the three strains grown on 0.1BHI soft agar (0.2%). Values are mean ± standard deviation of three experiments with independent precultures. For each preculture and strain at least three growth zones were measured. Statistical analysis was performed by ANOVA with Dunnett's multiple comparisons test with *L. monocytogenes* EGD‐e WT set as control condition

Besides *fliQ* and *motA*, three other genes (*flaA*, *fliD*, and *fliI*) involved in flagellar motility and its regulation were shown to impact on biofilm formation by the transposon mutant screen (Alonso et al., 2014). Flagellar motility has previously been shown to play a role in adhesion and biofilm formation of *L. monocytogenes* (Di Bonaventura et al., 2008; Lemon, Higgins, & Kolter, 2007; Todhanakasem & Young, 2008). Interestingly, 16 of the 44 genes *lmo_0675‐lmo_0718* of *L. monocytogenes* EGD‐e that encode for the flagellar apparatus were differentially regulated in the Δ*agrD* mutant (Supplementary File S1). Although these genes show divergent expression (i.e., some are up‐ and others down‐regulated) in the mutant, we performed motility assays to test if this strain shows altered expression or functionality of flagella. However, no difference in swimming motility was observed between the Δ*agrD* mutant and the WT or complemented strain at 25°C on 0.1BHI plates containing 0.2% (w/v) agar (Figure 3).

**Figure A1:**
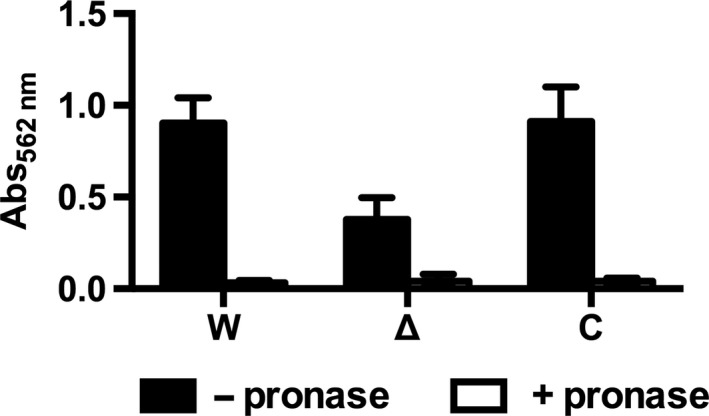
Sensitivity of biofilms of *L. monocytogenes* EGD‐e WT (W), EGD‐e Δ*agrD* (Δ), and EGD‐e Δ*agr*D::pIMK2*agrD* (C) to pronase. Biofilms were grown in BHI or 0.1BHI at 25 or 37°C in the absence (black bars) or presence (white bars) of 1 mg/ml pronase. Biofilm biomass was quantified by crystal violet staining and measuring absorbance at 562 nm (Abs_562nm_) after 24 hr of growth in polystyrene microtiter plates. All values are mean ± standard deviation of three independent experiments

In the absence of other indications about the possible reason for the phenotype of *L. monocytogenes* EGD‐e Δ*agrD*, we further analyzed the data set of genes differentially expressed in this strain. We reasoned that impaired attachment to the substratum of the mutant and interaction with other bacteria might be involved in the observed phenotype. These processes are mediated by proteins that are either secreted into the environment (exoproteins) or attached to the bacterial cell envelope. In fact, presence of pronase completely abolished biofilm formation of all three tested strains (Appendix Figure A1). Thus, we retrieved the cellular localization of all *agr*‐regulated proteins as annotated on the Listeriomics web page (https://listeriomics.pasteur.fr/Listeriomics/#bacnet.Listeria), which is based on an extensive in silico analysis (Renier, Micheau, Talon, Hébraud, & Desvaux, 2012). A total of 995 genes (34.8%) in the genome and 293 genes (i.e., 41.0%) of the *agr*‐regulated genes of *L. monocytogenes* EGD‐e encode for (predicted) extracytoplasmatic proteins (Table 2). Amongst the 715 *agr*‐dependent genes, 19 (2.7%) encode for exoproteins (i.e., proteins secreted and released into the extracellular environment), 25 (3.5%) for lipoproteins, 27 (3.8%) for cell wall proteins, 187 (26.2%) for integral membrane proteins, and 35 (4.9%) for cytoproteins (i.e., proteins predicted to be secreted via non‐classical pathways). None of the groups seems to be markedly overrepresented in the *agr*‐regulated genes. Nevertheless, the percentages of the *agr*‐regulated genes within these groups (except for exoproteins) were comparable or higher compared to the percentage of the respective group on the genome level suggesting that the *agr* system is involved in the regulation of biosynthesis, structure, and function of the cell envelope. Of note, the *agr*‐regulated genes included 10 genes for internalins or internalin‐like proteins, 15 genes for peptidoglycan‐associated proteins, and a number of genes for penicillin binding proteins and proteins with (know or presumable) cell wall‐hydrolyzing activity (Supplementary File 1). This indicates that the Δ*agrD* system is involved in regulation of cell envelope proteins that may be relevant for attachment to and interaction with abiotic surfaces as well as amongst bacterial cells.

**Table 2:** Number and percentage of different groups of genes encoding extracytoplasmatic proteins amongst the *agr*‐regulated genes of *L. monocytogenes*

Group of genes	No. (%) of genes in genome	No. (%) of *agr*‐regulated
All in genome^1^	2,587	715 (25.0)
Exoproteins^2^	80 (2.8)	19 (2.7)
Lipoproteins^2^	65 (2.3)	25 (3.5)
CW‐proteins^2^	56 (2.0)	27 (3.8)
IM‐proteins^2^	686 (24)	187 (26.2)
Cytoproteins^2^,^a^	108 (3.8)	35 (4.9)
All extracytoplasmatic proteins^2^	995 (34.8)	293 (41.0)

Information taken from: ^1^ *L. monocytogenes* EGD‐e genome on Listeriomics database (https://listeriomics.pasteur.fr/Listeriomics/#bacnet.Listeria); ^2^Renier et al. (2012).

^a^Cytoproteins are predicted to be secreted via non‐classical pathways.

**Figure 4:**
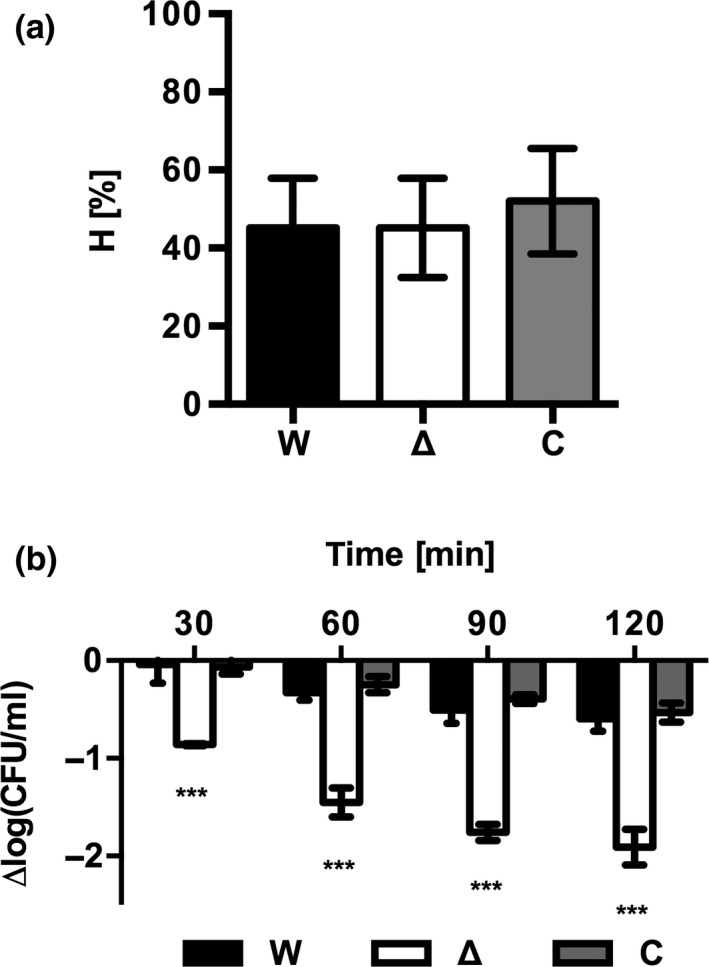
(a) Surface hydrophobicity and (b) resistance of *L. monocytogenes* EGD‐e WT (W), EGD‐e Δ*agrD* (Δ), and EGD‐e Δ*agr*D::pIMK2*agrD* (C) exposed to lysozyme (b). (a) Surface hydrophobicity (H [%]) was evaluated using MATH assay. (b) Bacteria from exponential growth phase were resuspended in 0.1BHI containing 5 µg/ml lysozyme and incubated for the indicated time. Changes in viability are reported as Δlog_10_(CFU/ml) compared to bacterial counts before treatment. Values are mean ± standard deviation of three independent experiments. Statistical analysis was performed by ANOVA with Dunnett's multiple comparisons test with *L. monocytogenes* EGD‐e WT set as control condition (***p < 0.001)

Altered surface protein profiles may result in changes in the physicochemical properties of the bacterial surface such as charge and hydrophobicity, which were shown to play a role in adhesion and biofilm formation of *L. monocytogenes* (Di Bonaventura et al., 2008; Takahashi, Suda, Tanaka, & Kimura, 2010). MATH assays performed in xylene revealed that *L. monocytogenes* EGD‐e Δ*agrD* did not differ in surface hydrophobicity compared to the WT or complemented strain when bacteria were grown in 0.1BHI at 25°C (Figure 4a). Similar results were obtained, when octadecene was used as solvent (data not shown).

Another functional consequence of an altered cell wall composition could be changes in the resistance to cell wall damage. To test this possibility, the resistance of *L. monocytogenes* EGD‐e Δ*agrD* to treatment with 5 µg/ml lysozyme was tested in 0.1BHI (Figure 4b). Under these conditions, viability of the WT and complemented strain decreased by about 0.5 logs during the first 120 min of lysozyme challenge. More importantly, the sensitivity of the Δ*agrD* mutant was significantly increased at any time point measured and viable counts were reduced by about 2 logs after 120 min.

Collectively, the obtained results suggest that the biofilm phenotype of *L. monocytogenes* EGD‐e Δ*agrD* is not a general feature of this mutant but is only relevant under specific conditions. The experimental conditions under which the mutant displays reduced biofilm formation include nutrient limitation and reduced osmolarity. These are the conditions similar to those encountered in difficult to access reservoirs in food processing plants (Carpentier & Cerf, 2011; Ferreira et al., 2014; da Silva & De Martinis, 2013). Thus, the *agr* system may be important for adaptation and survival of *L. monocytogenes* at such sites.

The observed phenotype of the Δ*agrD* mutant is not associated with differences in eDNA abundance, increased lysis in hypoosmotic conditions, flagellar motility, or surface hydrophobicity. It is more likely, that reduced biofilm formation of *L. monocytogenes* EGD‐e Δ*agrD* is the result of an altered cell envelope proteome, which manifests in reduced adhesion to the abiotic surface and/or to neighboring bacteria or the biofilm matrix and, in consequence, increased dispersal. The previously published transcriptional data (Riedel et al., 2009) provided first indications for genes and their products possibly involved in these phenotypes. In further studies, the contribution of these factors to the observed phenotype and their expression levels need to be investigated for example, by qPCR and experiments using knock‐out mutants of the respective genes.

## CONFLICT OF INTERESTS

The authors declare no conflict of interests.

## AUTHORS CONTRIBUTION

CU conceived the study. MZ, FIB, PC, DB, and LGG carried out experiments. PC, DB, AIN, GMS, and CUR analyzed data. DB, AIN, and CUR drafted the manuscript and all authors contributed to preparing the final version of the manuscript. All authors read and approved the final manuscript.

## ETHICS STATEMENT

Not required.

## DATA ACCESSIBILITY

All relevant data are presented in figures, tables, or in Supplementary File 1. Raw data used for preparation of figures will be made available on request. The original microarray data were deposited along with Supplementary File 1 on the publicly accessible data repository FigShare under the following https://doi.org/10.6084/m9.figshare.7577591.
